# Boosting Blood Donations Through Facebook Engagement: Randomized Controlled Field Trial

**DOI:** 10.2196/64740

**Published:** 2025-05-12

**Authors:** Steven Ramondt, Peter Kerkhof, Eva-Maria Merz

**Affiliations:** 1 Department of Donor Medicine Research Sanquin Research Amsterdam Amsterdam The Netherlands; 2 Department of Communication Science Vrije Universiteit Amsterdam Amsterdam The Netherlands; 3 Department of Sociolgy Vrije Universiteit Amsterdam Amsterdam The Netherlands

**Keywords:** blood donation attitudes and behavior, Facebook intervention, randomized controlled trial, blood banks, social media, behavior, public health, blood donation, Facebook, promotion

## Abstract

**Background:**

Social media platforms have shown considerable potential in shaping behaviors and have become a central component of public health and organizational outreach efforts. Blood collection agencies increasingly rely on social media not only for donor recruitment and retention but also for promoting donation-related behaviors. Regular, day-to-day status updates form a significant part of the communication strategies implemented by blood banks.

**Objective:**

Despite social media’s promise as a tool for behavior change, evidence supporting the persuasive effects of routine day-to-day status updates remains limited—not only within the context of blood donation but also across broader health domains. To address this gap, we examine long-term attitudinal and behavioral outcomes to understand better the impact of an organization’s social media efforts on health-related behaviors.

**Methods:**

We conducted a randomized controlled field trial to investigate the effects of a blood bank’s Facebook page on donation attitudes and behavior. All newly registered blood donors were invited to participate, and a total of 1891 participants were randomly assigned to either the experimental or control group. Participants were randomly assigned to either follow a blood bank’s Facebook page or not. The study used a 2 (new Facebook followers vs non-Facebook followers) × 2 (premeasure vs postmeasure) mixed design, with an additional observational arm consisting of current Facebook followers (n=415). Outcomes were measured at 2 and 12 months following participation, incorporating both between- and within-participant comparisons.

**Results:**

After 1 year, no significant interaction effects (group × time) were found for any of the attitudinal variables, including attitudes toward blood donation, intention to donate, attitudes toward the blood bank, perceived warmth and competence, contemplation of donorship, or contemplation of the blood bank. The experimental group was 32% more likely to have made a first donation (odds ratio [OR] 1.32, 95% CI 1.01-1.73) compared with the control group. Additionally, the experimental group made 12% more whole blood donations (incidence rate ratio [IRR] 1.12, 95% CI 1.01-1.24) and 17% more total donations (IRR 1.17, 95% CI 1.06-1.28) after 1 year than the control. No significant effects were observed for the observational group compared with the control group in terms of either whole blood donations or the total number of donations. Women were significantly more likely to donate more frequently (IRR 1.97, 95% CI 1.80-2.16) and less likely to discontinue their donor career (OR 0.52, 95% CI 0.37-0.72). Furthermore, older participants were slightly more likely to donate (OR 1.01, 95% CI 1.00-1.02).

**Conclusions:**

Our study provides initial evidence that regular engagement with a Facebook page can positively influence behavior in ways that offer meaningful benefits to organizations, including blood banks. While attitudinal effects appear limited, our findings demonstrate that social media efforts can nonetheless drive significant behavioral outcomes. Finally, we offer practical insights and actionable recommendations that blood banks and similar organizations can adopt to replicate these results and encourage prosocial behavior.

**Trial Registration:**

OSF Registries 10.17605/OSF.IO/2QGSU; https://osf.io/2qgsu

## Introduction

Blood-derived products have a profound impact on global health, serving as a critical component of modern health care systems [1]. According to the World Health Organization, millions of units of blood and plasma are collected worldwide each year to meet the ever-growing demand for these life-saving products [2]. In Europe alone, more than 25 million units of blood are collected annually, benefiting over 4 million patients [3,4]. Ensuring a safe and adequate supply of blood-derived products is therefore essential for effective patient care, improved health outcomes, and life-saving interventions globally. Remarkably, only 2%-3% of the population in Western countries typically contributes to this vital resource, making the stimulation and retention of a sufficient donor pool imperative [3]. Previous research has shown that retaining existing donors is both more cost-effective and safer than recruiting new ones [5-7]. Thus, efforts to encourage repeat donations from loyal donors are of paramount importance.

The advent of social media platforms has provided a valuable avenue for promoting blood donation and addressing challenges related to maintaining an adequate blood supply [8]. Research has shown that platforms such as Facebook can be effective in raising awareness about blood donation and encouraging donation behaviors [9,10]. These platforms offer unique opportunities to engage with potential donors, disseminate compelling narratives, remove barriers to donation, and foster a sense of community involvement [8,11-13]. Elements such as peer-to-peer communication, social networking, and social influence are leveraged through social media to encourage donation practices [10,14]. Moreover, the accessibility and broad reach of these platforms make them effective tools for engaging diverse populations and promoting blood donation across various regions and countries [10]. Thus, social media holds significant promise for raising awareness, encouraging donation behaviors, and addressing the challenges associated with maintaining an adequate blood supply.

Social media have become the cornerstone of many public health and agency efforts [15,16] and have been shown to be effective, low-cost recruitment tools in the health domain [17,18]. Like most nonprofit organizations, blood banks have adopted social media to support their mission [11,19-21]. Guo and Saxton [22] delineated a 3-tiered framework, in their pyramid model of social media-based advocacy, to explain how nonprofits garner support for their cause through social media. In stage 1, nonprofit organizations focus on *expanding their audience reach.* Concurrently, they endeavor to sustain engagement by *keeping the flame alive*—leveraging social media to foster interest and cultivate positive attitudes toward their causes (stage 2). In addition, they *step up to action* and utilize these platforms to bolster intent and participation through targeted calls to action (stage 3) [22]. For blood banks, garnering support through social media is often realized through regular day-to-day status updates, which constitute a significant portion of their overall social media efforts [8,11]. A thorough examination of these activities is therefore crucial for effective donor management.

Although there is ample evidence that following organizations’ social media accounts is associated with more positive attitudes toward those organizations (eg, [23]), evidence that this is due to the persuasive effects of daily social media status updates—rather than self-selection based on already positive attitudes—remains scarce. Studies that allow for causal conclusions have been conducted almost exclusively in the context of business and marketing, focusing on sales and engagement as dependent variables. In the business domain, a recent meta-analysis [24] concluded that an active social media presence by brands positively affects both engagement and sales.

Despite the acknowledged importance of social media in the field of blood donation, only 1 recent study has tested its effects on donation behavior using a design that allows for causal conclusions. Harrell et al [9] examined the impact of the Facebook blood donation tool—which connects users to nearby donation locations—by comparing blood donation data across areas with different rollout dates of the tool. Their study found a 4.0% increase in total donations and an 18.9% increase in first-time donations attributable to the Facebook donation tool.

Our study focuses on understanding the impact of organizations’ social media page efforts on donation attitudes and behavior among newly registered donors, as recorded by the blood bank. To this end, we use Facebook—the predominant social media platform used by Dutch blood banks [8,11]. Following the protocol by Beukeboom et al [25], we use experimental manipulation to address current research limitations and discern the nature of the observed effects. In this manipulation, new donors who have just registered are randomly assigned to either follow a blood bank’s Facebook page or not. An additional observational arm includes new donors who already follow the blood bank’s Facebook page. This design allows us to determine whether the observed effects stem from a selection effect or are the result of a causal effect due to exposure to Facebook posts. In addition, it enables the evaluation of social media’s effectiveness within 2 critical phases of the pyramid model of social media–based advocacy for persuasion: stage 2, “Keeping the flame alive,” and stage 3, “Stepping up to action.” This evaluation is conducted by examining attitudes (stage 2), behavioral intentions (stage 3), and actual blood donation behaviors (stage 3)—key constructs of the dominant behavioral theory in blood donation, the Theory of Planned Behavior [26]. Blood donation attitudes and behavioral intentions are crucial factors for donor retention and have been shown to predict repeat blood donations [27-30]. Additionally, we assess these stages by evaluating donors’ contemplation of blood donation and the blood bank (stage 2), alongside 2 dimensions that critically shape organizational perceptions: social judgments of warmth and competence (stage 3). High levels of perceived organizational warmth and competence can enhance the willingness to engage and reduce obstacles that might otherwise impede action [31]. By examining these factors, we aim to evaluate the platform’s efficacy not only in sustaining donors’ current relationships with blood donation and the blood bank but also in promoting ongoing engagement and boosting future donations.

Work by Beukeboom et al [25] highlighted how social media engagement, such as liking and following a brand on Facebook, can positively impact brand evaluations and purchase intentions, suggesting that virtual interactions on social media can translate into actual liking and increased intention. Given that current followers of the blood bank’s Facebook page have already been exposed to the content, we hypothesize the following:

- Hypothesis 1: Newly registered blood donors who followed the blood bank’s Facebook page before registration (hereinafter “current followers”) will exhibit more positive attitudes toward the blood bank at the initial measurement (T0) compared with both groups of newly registered donors—those assigned to follow the blood bank’s Facebook page (hereinafter “new followers”) and those not assigned to follow the page (hereinafter “nonfollowers”).

Beukeboom et al [25] provided initial evidence of the effect of linking to a brand page on brand attitudes and intentions over a 1-month period. However, it is plausible that the efficacy of organizational content is strongest when it is new, with effects potentially diminishing over time. To address this, we extend the study over a longer duration, examining the impact of exposure to Facebook posts over 2- and 12-month periods. Additionally, we expect to observe changes only in participants for whom the situation changes—that is, those who are newly exposed to the Facebook page. Therefore, we hypothesize that:

- Hypothesis 2. Both current and new blood bank Facebook followers will exhibit more positive attitudes toward the blood bank, more favorable attitudes toward blood donation, and higher intention to donate blood compared with nonfollowers at postmeasurements (T1, after 2 months, and T2, after 12 months).
- Hypothesis 3. New blood bank Facebook followers will show an increase in their attitudes toward the blood bank between pre- and postmeasures (T1 and T2), whereas attitudes toward the blood bank from current followers and nonfollowers will remain unchanged.

This study further aims to build on previous research by examining the impact of social media on behavior. We analyze retention behavior and donation frequency, with a particular focus on the crucial first donation. First donations are a strong predictor of long-term donation behavior, overall donation frequency, and the likelihood of discontinuation in donor careers [32-34]. Retaining newly recruited donors is a significant challenge for blood banks: around 24% of Dutch donors do not make a second donation [32,35]. This challenge may be even more pronounced in other regions, as evidenced by return rates after the first donation in England, North Wales, and Iran (52%), and Ohio (35%) [36-38]. In the Netherlands, where only 1 organization (Sanquin) is authorized to collect and distribute blood and blood components, newly registered blood donors must meet specific health criteria before donation, a process verified through a New Donor Examination (NDE). This examination includes a health questionnaire reviewed by a physician and a blood test for transfusion-transmissible infections. Approximately 82% of newly registered donors attend the NDE. Following successful testing and clearance, donors are permitted to proceed with their first blood donation, with 63% of them donating (77% of those who underwent the NDE). Because of the persuasive nature of the blood bank’s Facebook posts, we hypothesize that exposure to these posts will lead to the following:

- Hypothesis 4. New and current blood bank Facebook followers will be more likely to show up for their first blood donation (both the NDE and the regular first donation) compared with nonfollowers.
- Hypothesis 5. New and current blood bank Facebook followers will have donated more frequently compared with nonfollowers after 1 year.

Furthermore, in light of the blood bank’s social media communications, which aim to mitigate the high donor attrition rates observed in blood donation, it can be posited that the repeated emphasis on the importance of the donor community and the impact of blood donation on patients [3,11,39] may contribute to a reduction in donor attrition. This leads to the following hypothesis:

- Hypothesis 6. New and current blood bank Facebook followers will be less likely to have ceased their donor careers compared with nonfollowers after 1 year.

## Methods

### Study Overview and Design

**Figure 1:**
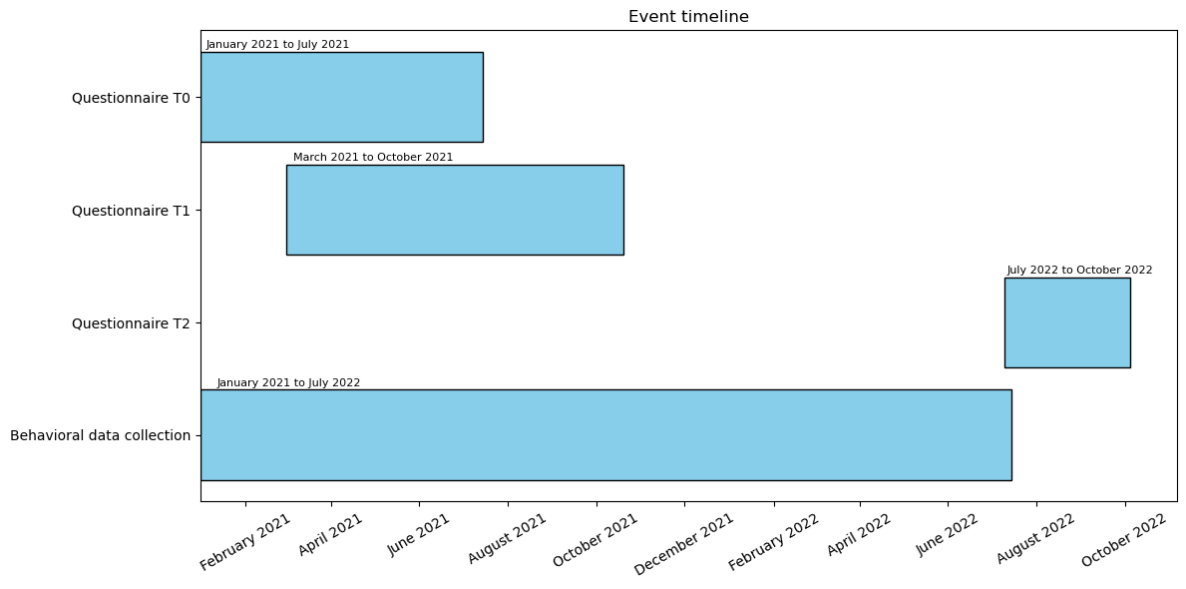
Timeline for data collection. This figure outlines the timeline of data collection from the Dutch blood collection agency study, starting with initial participant recruitment upon donor registration. The timeline includes three questionnaires: the baseline questionnaire administered immediately after registration (T0), a follow-up at two months (T1) to assess changes in attitudes and Facebook interactions, and a final questionnaire at twelve months (T2) to evaluate long-term attitudinal shifts. Simultaneously, behavioral data regarding actual donations were extracted from the agency's database to correlate with survey responses.

We conducted a randomized controlled field trial in which we invited all newly registered blood donors to participate in our study on the effects of the Dutch blood bank’s Facebook page (see Figure 1 for the timeline). The blood bank’s primary recruitment method is through social media, and it maintains an active social media presence, with its Facebook page having approximately 78,000 followers at the time of the study. About 4 posts are published weekly, focusing on the 5 social media communication pillars of the blood bank: Donor Community, Better Patient Life, Inside the Blood Bank, Blood, and Plasma.

- Donor Community: Highlighting stories and experiences of blood donors to foster a sense of community and encourage others to donate.
- Better Patient Life: Sharing testimonials and success stories from patients who have benefited from blood donations, illustrating the direct impact donors have on individuals’ lives.
- Inside the Blood Bank: Providing behind-the-scenes glimpses into the daily operations of the blood bank and the processes involved in blood collection and distribution.
- Blood: Educating the audience about the importance of blood donation, blood types, and dispelling common myths associated with the donation process.
- Plasma: Informing followers about plasma donation, its uses in medical treatments, and the specific need for plasma donors.

### Study Procedure

Following the preregistered protocol (see the “Trial Registration” section), participants were randomly assigned to either the experimental or the control group. This 2 (new Facebook followers and non-Facebook followers) × 2 (premeasure and postmeasure) mixed design, with an additional observational arm (current Facebook followers of the blood bank’s page), varied between and within participants, with results measured at 2 and 12 months after participation.

**Figure 2:**
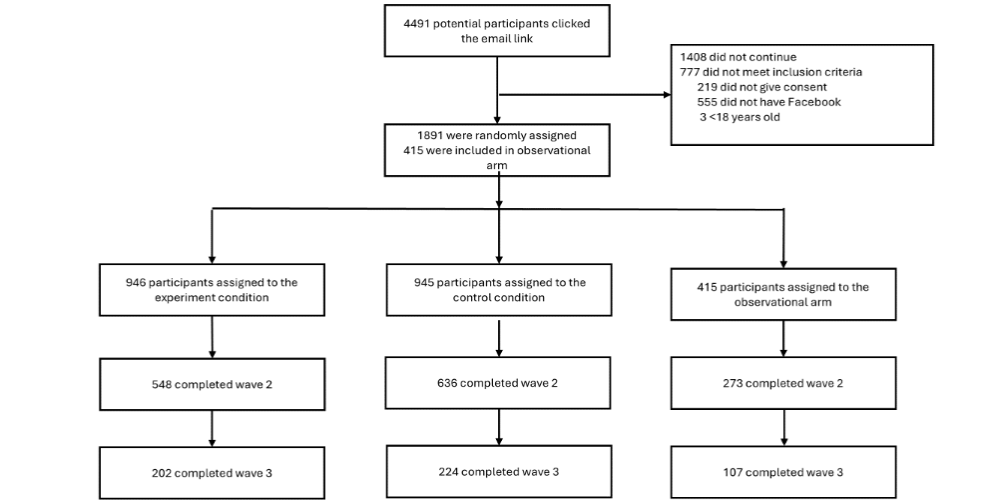
Flow diagram.

Participants were recruited from the database of the Dutch blood collection agency immediately after registering as new donors. They were invited to participate via email, which included a direct online link to the study, sent within 2 weeks of signing up to become blood donors. To participate in the study, individuals had to be within the donation age range (18-65 years at the time of enrollment) and have an active Facebook account (see Figure 2 for the flow diagram). Upon clicking the link, participants were directed to an online consent form. After giving consent and joining the study, participants completed several questions about their Facebook usage, as well as attitudinal questions regarding blood donation and the blood bank. They were then asked about their familiarity with the blood bank’s Facebook page. Participants who were familiar with and currently liked the page were assigned to the observational arm. Those who were not currently liking the Facebook page were randomly assigned to either the experimental or control group through an automated process in the online software Qualtrics (SAP SE).

Next, only participants assigned to the experimental group were prompted via an online text to like the blood bank’s Facebook page. They were provided with instructions and a direct link to do so immediately. Afterward, participants were asked whether they liked the page. Finally, demographic information was collected, and participants were asked to provide their email addresses, allowing us to contact them for the second (after 2 months) and third (after 12 months) waves of questionnaires. During waves 2 and 3, participants were only asked attitudinal questions about blood donation and the blood bank; no demographic or familiarity questions were collected.

### Constructs and Measures

We examined attitudes and behavioral intentions toward blood donation, key concepts of the Theory of Planned Behavior, which have previously been shown to be the most critical components for blood donor retention [29,30]. The key constructs and measures are described below (see Multimedia Appendix 1 for the questionnaire). Questionnaire variables were collected for all 3 waves unless specified otherwise.

### Attitudinal Outcomes

#### Blood Donation Intention

The extent to which an individual intends to donate blood was measured using 2 items [40] on a 5-point Likert scale, ranging from “1=completely disagree” to “5=completely agree.” An example item is “I intend to donate blood within the next 12 months.” The items were compiled into a mean index (Cronbach α=0.85).

#### Blood Donation Attitude

Six items measured attitude toward blood donation on a 5-point Likert scale, based on [40]. The question stem was “For me, donating blood in the next 12 months would be...,” (eg, “1=useless” to “5=useful”). The items were compiled into a mean index (Cronbach α=0.70).

#### Blood Bank Attitude

Attitude toward the blood bank was measured with 4 questions on a 9-point Likert scale, based on Beukeboom et al [25]. The question stem was: “Please rate [organization]...bad – good, not nice – nice, attractive – unattractive, and qualitatively bad – qualitatively good.” The items were compiled into a mean index (Cronbach α=0.86).

#### Warmth and Competence

Perceptions about the warmth and competence of the blood bank were measured with 6 items (3 for each construct) on a 7-point Likert scale [31], ranging from “1=completely disagree” to “7=completely agree.” An example is “I find that [organization] is warm.” The items were compiled into mean indices (Cronbach α=0.87 for warmth and Cronbach α=0.90 for competence).

#### Contemplation

Participants were asked how often they thought about blood donation and the blood bank with 2 questions, rated on a 9-point Likert scale, ranging from “1=never” to “9=multiple times a day.” For example, “How often do you think about blood donation?”

### Behavioral Outcomes

#### Assessment of Donation Behavior

Blood donation behavior was assessed by monitoring actual blood donations, which were retrieved from the blood bank registry. Each visit to the blood bank that resulted in blood collection was systematically recorded, including NDEs. For this study, all recorded donations from participants were included in the analysis, covering a 1-year period following their initial participation in the study.

#### First Donation

Every individual registering at Sanquin in The Netherlands is first invited for an NDE. This visit includes a blood sample to screen for infectious diseases, assess blood type, and check for potential antibodies. If all criteria are met, prospective donors are invited for their first donation a few weeks later. Both of these blood withdrawals are separately recorded. In our analyses, we include attendance (yes/no) for both events.

#### Blood Donation

Donation intervals and invitations may vary depending on the donation type. For example, plasma donations can occur every 2 weeks. Therefore, we distinguish between the frequency of total whole blood donations and the total number of blood donations (including nonwhole blood donations) made in the year following participation in this study.

#### Donation Cessation

Using blood donation records from the blood bank registry, we investigated whether participants ceased making donations during the year following their participation in the study (yes/no).

### Control Variables

#### Overview

Age, education, and sex were measured at baseline and included as control variables in the behavioral analyses, as these factors could influence the overall potential for blood donations. For donor health reasons, females can donate whole blood 3 times a year, while males are allowed up to 5 donations annually in the Netherlands [41]. Additionally, age affects donation requirements, such as ferritin levels [41,42]. The following variables related to general social media use were only measured during the premeasure period and were included as control variables if significant differences were observed at baseline, as stated in the preregistration.

#### General Facebook Time

Time spent on Facebook was measured with a single item based on Beukeboom et al [25]. Participants were asked, “How often do you spend time on Facebook?” on a 12-point Likert scale ranging from “1=never” to “2=multiple times an hour.”

#### General Facebook Intensity

The intensity of Facebook usage [43] was assessed using 6 items rated on a 5-point Likert scale ranging from “1=completely disagree” to “5=completely agree.” Sample items included, “Facebook is part of my everyday activity.” The items were compiled into a mean index (Cronbach α=0.88).

The following variables related to social media use and blood donation were measured only at wave 2 (T1) and wave 3 (T2) and were included as control variables.

#### Blood Bank Social Media Channels

Participants were asked whether they followed other social media channels (ie, Twitter and Instagram) of the blood bank. Two dummy variables (yes/no) were created based on the question “Do you follow the Twitter/Instagram page of the blood bank at this moment?”

#### Contact and Donation

Participants indicated whether they had been contacted for a blood donation appointment and whether they had donated since participating in the study.

### Analytic Approach

ANOVAs with Bonferroni correction for multiple comparisons and chi-square tests were used to examine mean differences between experimental groups and the observational arm on demographic variables, contact, and donation at baseline. As a result of violations of the assumptions of normality and homoscedasticity (equal variances), nonparametric Kruskal-Wallis tests were conducted on attitudes, intention, and control variables at baseline. The main attitudinal outcome differences between the experimental groups at 2 and 12 months after participation were assessed using mixed ANOVAs on the dependent variables. Poisson regression analyses were conducted for the number of donations, and logistic regression models were used for binary behavioral outcomes. All analyses were performed using R version 4.3.0 (R Foundation for Statistical Computing).

### Ethics Approval

The Sanquin Institutional Review Board approved the study protocol. All participants provided signed informed consent. To ensure privacy and confidentiality, all collected data were deidentified. Participants did not receive any compensation.

## Results

### Manipulation Check

Following the preregistered protocol, we checked whether the experimental group manipulation was successful. Participants who were not exposed to the Facebook page were excluded from the analyses; 155 out of 548 (28.0%) participants assigned to the experimental condition declined to like the blood bank’s Facebook page when prompted during the baseline measure. Common reasons included a lack of interest, not being active on or not wanting to use Facebook, and hesitations about joining an organizational Facebook page immediately after registering as a blood donor (and before having donated).

Postmeasure checks also revealed that 140 participants out of 636 participants (22.0%) assigned to the control condition reported having started following the blood bank’s Facebook page during the 2-month follow-up period. These participants were excluded from the analyses. Figure 2 presents the study flowchart (also see Multimedia Appendix 2).

### Control Analyses

Table 1 presents the results of 1-way ANOVAs comparing the observational, experimental, and control groups. A significant difference was found between the groups in terms of age (*F*_2,1159_=36.61, *P*<.001). Tukey post hoc comparisons revealed no significant age difference between the control and experimental groups (*P*=.99). However, participants in the observational arm are significantly older than those in both the experimental (*P*<.001) and control groups (*P*<.001). No significant differences between groups were found for sex (*χ*^2^_2_=0.57, *P*=.75) or prior donation status (*χ*^2^_2_=0.41, *P*=.81).

**Table 1:** Sample characteristics and comparison of all participants measured at baseline.

Characteristics		Group				*P* value		
		Experimental (n=393)^a^	Control (n=496)^a^	Observational (n=273)^a^	Experimental vs control		Experimental vs observational	Control vs observation
**Demographics**								
	Age	32.75 (12.77)	32.64 (13.76)	40.54 (13.00)	.99		<.001	<.001
	Female, n (%)^b^	297 (75.6)	365 (73.6)	206 (75.5)	>.99		>.99	>.99
**Control variables**								
	General Facebook time (1-12)^c^	7.82 (2.15)	7.61 (2.26)	8.83 (1.54)	.110		<.001	<.001
	Facebook intensity (1-5)^c^	2.57 (0.85)	2.44 (0.88)	3.09 (0.80)	.025		<.001	<.001
	Has donated, n (%)^b^	146 (55.5)	184 (55.4)	107 (58.2)	>.99		>.99	>.99
	Has appointment, n (%)^b^	264 (79.0)	335 (79.2)	185 (87.7)	>.99		>.99	.02
**Dependent variables**								
	Attitude toward blood donation (1-5)^c^	4.26 (0.43)	4.17 (0.43)	4.28 (0.48)	.003		.26	<.001
	Intention toward blood donation (1-5)^c^	4.36 (0.81)	4.29 (0.82)	4.42 (0.77)	.10		.36	.02
	Attitude toward blood bank (1-9)^c^	7.49 (1.02)	7.33 (1.12)	7.59 (1.08)	.09		.14	.005
	Warmth (1-7)^c^	5.59 (0.84)	5.44 (0.92)	5.59 (0.99)	.03		.59	.030
	Competence (1-7)^c^	5.85 (0.90)	5.73 (0.94)	5.76 (1.06)	.09		.35	.35
	Contemplation donorship^c^	4.08 (1.32)	4.04 (1.16)	3.96 (1.35)	.77		.24	.24
	Contemplation blood bank^c^	3.36 (1.62)	3.32 (1.65)	3.83 (1.68)	.66		.001	<.001

^a^Values are presented as mean (SD) unless indicated otherwise.

^b^Difference with chi-square.

^c^Difference with Kruskal-Wallis.

Five participants in both the observational and experimental arms followed the blood bank on Twitter. In the observational arm, 29 participants followed the blood bank on Instagram, compared with 35 in the experimental arm and 12 in the control arm. Following on Twitter and Instagram represented only 5 out of 889 (0.56%) and 47 out of 889 (5.28%) participants, respectively, included in the experimental and control conditions, and these were therefore excluded from further analyses. Post hoc comparisons showed no differences between the experimental and control conditions for any other control variables, and these were therefore not included as covariates, as stated in the preregistration. The observational arm scored higher on general Facebook time (*χ*^2^_2_=31.82, *P*<.001), Facebook intensity (*χ*^2^_2_=52.45, *P*<.001), and having an appointment to donate (*χ*^2^_2_=7.84, *P*=.02) compared with both the control and experimental groups (Table 1).

### What Are the Attitudinal Differences Between Groups at the Baseline?

In line with hypothesis 1, the observational arm, consisting of the current follower group, shows more positive attitudes toward blood donation (*χ*^2^_2_=17.52, *P*<.001) and the blood bank (*χ*^2^_2_=10.47, *P*=.005), and contemplates blood donation more frequently than participants in the other 2 groups (*χ*^2^_2_=16.76, *P*<.001; see Table 1). In addition, participants in the observational arm scored higher on the intention to donate compared with the control group (*χ*^2^_2_=7.80, *P*=.02). No differences between the groups were found regarding the blood bank’s competence (*χ*^2^_2_=4.83, *P*=.09) and contemplation of donation (*χ*^2^_2_=32.48, *P*=.29). Participants in the control group scored lower on attitude toward blood donation compared with both the observational (*P*=.006) and experimental groups (*P*=.01). Similarly, participants in the control group scored lower on warmth (*χ*^2^_2_=8.52, *P*=.01) compared with participants in both the observational (*P*=.03) and experimental groups (*P*=.03).

### What Are the Short- (2 Months) and Long-Term (12 Months) Effects on Attitudes?

Overall, there are short-term (after 2 months) interaction effects (group × time) on attitude toward blood donation (*F*_2,1159_=7.90, *P*<.001) and warmth (*F*_2,1159_=4.05, *P*=.02; see Table 2). Attitude toward blood donation also showed a short-term group effect (*F*_2,1159_=8.16, *P*<.001). Bonferroni comparisons show that the attitudes of the experimental group decreased more compared with the other groups (*P*<.001). While the groups responded differently over time for warmth, as indicated by the interaction effect, there is no main group effect for warmth (*F*_2,1159_=2.05, *P*=.13), suggesting that the groups were not different. There was a short-term time effect for warmth (*F*_2,1159_=166.07, *P*<.001), with warmth decreasing for all groups. No short-term interaction effects were found for intention to donate (*F*_2,1159_=0.26, *P*=.77), attitude toward the blood bank (*F*_2,1159_=2.74, *P*=.06), competence (*F*_2,1159_=2.16, *P*=.11), contemplation of donorship (*F*_2,970_=.33, *P*=.72), and contemplation of the blood bank (*F*_2,967_=2.32, *P*=.10). These short-term findings do not support hypothesis 2, as both current and new Facebook followers did not exhibit more positive attitudes or higher intentions to donate blood compared with nonfollowers. Similarly, the data did not support hypothesis 3, as new Facebook followers did not show an increase in positive attitudes toward the blood bank between pre- and postmeasurements, and attitudes among current followers and nonfollowers remained unchanged.

**Table 2:** Main attitudinal outcomes. This table summarizes the key attitudinal outcomes measured across the study timeline. It includes data from baseline, 2 months, and 12 months after enrollment, comparing shifts in attitudes toward blood donation, the blood bank, and related behavioral intentions among different participant groups.

Dependent variables	Experiment				Control				Observational		
	Pre, mean (SD)	2 months (n=393), mean (SD)	12 months (n=151), mean (SD)	Pre, mean (SD)		2 months (n=496), mean (SD)	12 months (n=158), mean (SD)	Pre, mean (SD)		2 months (n=273), mean (SD)	12 months (n=107), mean (SD)
Attitude toward blood donation	4.26 (0.43)	4.16 (0.40)	4.22 (0.37)	4.17 (0.43)		4.19 (0.45)	4.19 (0.47)	4.28 (0.48)		4.31 (0.48)	4.28 (0.47)
Intention toward blood donation	4.36 (0.81)	4.22 (0.86)	4.26 (0.73)	4.29 (0.82)		4.17 (0.87)	4. 22 (0.79)	4.42 (0.77)		4.33 (0.81)	4.36 (0.89)
Attitude toward blood bank	7.49 (1.02)	6.98 (1.23)	7.13 (1.19)	7.33 (1.12)		7.00 (1.23)	7.06 (1.30)	7.59 (1.09)		7.25 (1.39)	7.33 (1.33)
Warmth	5.59 (0.84)	5.03 (0.98)	5. 07 (0.92)	5.44 (0.92)		5.09 (0.97)	5. 08 (1.01)	5.59 (0.99)		5.18 (1.14)	5.16 (1.06)
Competence	5.85 (0.90)	5.33 (1.07)	5.37 (1.02)	5.73 (0.94)		5.38 (1.03)	5.36 (1.06)	5.76 (1.07)		5.37 (1.18)	5.41 (1.18)
Contemplation donorship	4.08 (1.32)	3.83 (1.21)	3.90 (1.37)	4.04 (1.16)		3.82 (1.27)	3.75 (1.27)	3.96 (1.35)		3.80 (1.39)	3.72 (1.41)
Contemplation blood bank	3.31 (1.62)	3.95 (1.46)	3.94 (1.35)	4.17 (0.43)		4.19 (0.45)	3.71 (1.26)	4.28 (0.48)		4.31 (0.48)	4.18 (1.40)

There is a short-term time effect on all dependent variables (intention to donate: *F*_2,1159_=14.27, *P*<.001; attitude toward the blood bank: *F*_2,1159_=110.01, *P*<.001; competence: *F*_2,1159_=131.62, *P*<.001; donorship contemplation: *F*_2,970_=21.57, *P*<.001; and contemplating the blood bank: *F*_2,967_=91.60, *P*<.001), except for attitude toward blood donation (*F*_2,1159_=1.25, *P*=.26), with a decrease observed for all groups. Furthermore, there are short-term group effects on intention to donate (*F*_2,1159_=4.39, *P*=.01), attitude toward the blood bank (*F*_2,1159_=5.28, *P*=.003), and contemplating the blood bank (*F*_2,967_=7.42, *P*<.001). Intention to donate and blood bank contemplation decreased more rapidly for both the experimental and control groups compared with the observational group. Attitude toward the blood bank decreased the most for the experimental group and remained highest for the observational group. There is no main group effect for warmth (*F*_2,1159_=2.05, *P*=.13), competence (*F*_2,1159_=0.20, *P*=.81), or donorship contemplation (*F*_2,970_=0.35, *P*=.71).

After 1 year, none of the interaction effects (group × time) remained. The scores for attitude toward blood donation and all other measures converged to a more similar level across the groups (see Table 2): attitude toward blood donation: *F*_2,413_=1.63, *P*=.20; intention to donate: *F*_2,413_=0.51, *P*=.60; attitude toward blood bank: *F*_2,413_=0.09, *P*=.92; warmth: *F*_2,413_=0.06, *P*=.94; competence: *F*_2,413_=0.45, *P*=.64; donorship contemplation: *F*_2,321_=2.05, *P*=.13; and contemplating the blood bank: *F*_2,320_=1.73, *P*=.18.

All dependent variables show significant effects over time after 12 months: attitude toward blood donation: *F*_2,413_=26.45, *P*<.001; intention to donate: *F*_2,413_=41.83, *P*<.001; attitude toward blood bank: *F*_2,413_=56.70, *P*<.001; warmth: *F*_2,413_=129.19, *P*<.001; competence: *F*_2,413_=89.72, *P*<.001; donorship contemplation: *F*_2,321_=6.97, *P*=.009; and contemplating the blood bank: *F*_2,320_=29.15, *P*<.001. Bonferroni comparisons show a decrease in intention to donate, attitude toward the blood bank, warmth, and competence for all groups. However, there is an increase in contemplating the blood bank across all groups (see Multimedia Appendix 1 for cross-sectional analyses of the 2- and 12-month periods). In summary, the data did not support hypotheses 2 and 3, as the expected positive shifts in attitudes and intentions among new and current followers were not observed.

### What Are the Effects on Donation Behavior?

Contrary to hypothesis 4, new and current blood bank Facebook followers were not more likely to attend their NDE compared with nonfollowers (see Tables 3 and 4). However, the experimental group was 32% more likely to make their first donation (odds ratio [OR] 1.32, 95% CI 1.01-1.74) compared with the control group. Similarly, the experimental group made 12% more whole blood donations (incidence rate ratio [IRR] 1.12, 95% CI 1.01-1.24) and 17% more total blood donations (IRR 1.17, 95% CI 1.06-1.28) in a year compared with the control group, further demonstrating a positive effect of the intervention on donation frequency among new followers. However, no effects were found for the observational group compared with the control group in terms of both whole blood and total number of blood donations, contradicting hypothesis 5. In contrast to hypothesis 6, neither the experimental nor the observational group showed a lower likelihood of ceasing their donor career compared with the control group after 1 year. Regarding covariates, females made more donations in a year compared with males, both for whole blood (IRR 1.97, 95% CI 1.80-2.16) and for total blood donations (IRR 1.95, 95% CI 1.79-2.13). They were also more likely to donate for their NDE (OR 1.92, 95% CI 1.38-2.71) and to make their first donation (OR 2.11, 95% CI 1.59-2.81). Women were less likely to cease their donor career (OR 0.52, 95% CI 0.37-0.72). Age was positively related to donation behavior: being 1 year older increased the likelihood of making a first donation (OR 1.01, 95% CI 1.00-1.02) and of making more donations (IRR 1.01, 95% CI 1.00-1.01). No significant effects were found for education on the likelihood of making a first donation (OR 0.96, 95% CI 0.89-1.05) or making more donations (IRR 0.99, 95% CI 0.97-1.02).

**Table 3:** Main behavioral analyses. This table displays the outcomes of logistic regression analyses, presenting ORs^a^ and IRRs^b^ for key behavioral metrics.

Predictors	New donor examination			First donation			Whole blood donation			Total blood donation			Cease donation career	
	OR (95% CI)	*P* value	OR (95% CI)		*P* value	IRR (95% CI)		*P* value	IRR (95% CI)		*P* value	OR (95% CI)		*P* value
Intercept	1.36 (0.70-2.64)	.36	0.73 (0.40-1.32)		.30	1.21 (0.97-1.52)		.09	1.21 (0.98-1.50)		.07	0.74 (0.38-1.42)		.36
Age	1.00 (0.99-1.01)	.75	1.01 (1.00-1.02)		.007	1.00 (1.00-1.01)		.10	1.01 (1.00-1.01)		<.001	1.00 (0.99-1.01)		.75
Education	1.09 (1.00-1.20)	.05	0.96 (0.89-1.05)		.40	0.99 (0.96-1.02)		.62	0.99 (0.97-1.02)		0.65	0.91 (0.83-1.00)		.05
Female	1.92 (1.38-2.71)	<.001	2.11 (1.59-2.81)		<.001	1.97 (1.80-2.16)		<.001	1.95 (1.79-2.13)		<.001	0.52 (0.37-0.72)		<.001
Experiment	1.21 (0.89-1.64)	.22	1.32 (1.01-1.74)		.04	1.12 (1.01-1.24)		.03	1.17 (1.06-1.28)		.001	0.83 (0.61-1.12)		.22
Observational	1.17 (0.83-1.67)	.37	1.16 (0.85-1.59)		.36	1.09 (0.97-1.22)		.16	1.09 (0.97-1.02)		.11	0.85 (0.60-1.21)		.37

^a^OR: odds ratio.

^b^IRR: incidence rate ratio.

**Table 4:** Main behavioral outcomes. This table details the main behavioral outcomes of the study, reporting mean values with SDs and percentages. It compares these outcomes across the experimental, control, and observational groups to highlight differences in donation behavior during the study.

Dependent variables	Experiment (n=393)	Control (n=496)	Observational (n=273)
New donor examination (yes), n (%)	298 (75.8)	359 (72.4)	202 (74.0)
First donation (yes), n (%)	227 (57.8)	255 (51.4)	157 (57.5)
Whole blood donation, mean (SD)	1.75 (1.49)	1.59 (1.46)	1.75 (1.53)
Total blood donation, mean (SD)	2.08 (1.85)	1.81 (1.74)	2.06 (2.05)
Cease donation career, n (%)	95 (24.2)	137 (27.6)	71 (26.0)

## Discussion

### Principal Findings

This study investigated whether liking a blood bank’s Facebook page could enhance individuals’ donation attitudes, intentions, and, most importantly, donation behavior.

In line with hypothesis 1, we observed that participants who had already liked the blood bank’s Facebook page held more favorable attitudes toward the blood bank compared with both new followers and nonfollowers. These participants also expressed more positive views about blood donation and were more frequently engaged in thoughts about the blood bank. These findings align with earlier work (eg, [25,44]) and can be explained by both selection effects, wherein preexisting positive attitudes lead individuals to “like” the page, and causal effects resulting from exposure to the Facebook posts.

Our randomized field experimental design allowed us to draw causal inferences over 2- and 12-month periods. While the observational group exhibited more positive attitudes toward blood donation and the blood bank, higher intention to donate, and more frequent thoughts about the blood bank after 2 months compared with the control group, the experimental group did not show these differences. In contrast to hypotheses 2 and 3, we did not find any differences between the groups in warmth, competence, or thoughts about blood donation. Overall, exposure to the blood bank’s Facebook posts did not lead to sustained attitudinal changes. In fact, all attitudinal variables showed only minimal decline over time for all groups. The only exception was an increase in contemplation about the blood bank (and blood donation for the control group, which had started from a significantly lower baseline), observed across all groups.

This divergence from the short-term (ie, 1 month) effects on brand evaluation found by Beukeboom et al [25] suggests that such effects may diminish over extended periods. Clearly, the specific content participants are exposed to and the way in which an organization presents itself on Facebook play a crucial role and can significantly influence these outcomes. Nevertheless, this study found no immediate causal effect of Facebook exposure on attitudinal variables. Baseline attitudes were already high, with averages nearing the maximum. It is plausible that the elevated attitudes observed around donor sign-up are not sustainable, and the relatively nonintrusive act of liking a Facebook page may not be enough to counteract the natural decline over time. Despite the observed decline, all attitudinal variables remained at high levels, suggesting that the decrease is unlikely to have a significant impact. The universal increase in thoughts about the blood bank could be attributed to its prominent and favorable visibility during the COVID-19 crisis [45]. Another contributing factor could be the regular donation process itself, including receiving invitations and making actual donations, which all groups underwent.

Regarding behavioral outcomes, we hypothesized that both the experimental and observational groups would be more likely to attend their first blood donation session, donate more frequently, and be less likely to discontinue their donor careers compared with the control group, which did not follow the blood bank on Facebook (hypothesis 4-hypothesis 6). Consistent with our hypotheses, we observed significant differences in the rates of first-time donations, whole blood donations, and total donations between the experimental group and the control group. However, no differences were found between the observational group and the control group for any of the assessed behavioral outcomes. This suggests that while initial effects were evident after 1 year, the impact of Facebook exposure may diminish over time. The Facebook-following group was 32% more likely to have made a first donation—considering a critical indicator of subsequent donation behavior, overall donation frequency, and donor career termination [32-34]—compared with the control group. The period for observing donation cessation after the intervention was relatively brief. Given the observed increase in initial donations, it is conceivable that cessation effects might become more evident with a prolonged intervention period. Notably, the group subjected to the experimental intervention exhibited a higher rate of whole blood donations and overall donation activity compared with the control group. In 2022, the Netherlands reported a total of over 730,000 donations from slightly more than 406,000 donors. With an average of 1.7 donations per donor, a 17% increase in donations across potentially the majority of the donor population is significant. The typically short duration of donor engagement highlights the need to recruit 70,000 new donors in 2022 (with a target of 92,000, which was surpassed) to sustain a donor base comparable to the previous year’s total of just over 384,000 [46]. Although the operational complexities of blood banks are multifaceted, even modest increases in contributions from the existing donor pool can lead to a significant reduction in the demand for new donors. This indicates that even incremental improvements in donation frequencies, driven by social media, can have disproportionately positive effects on minimizing the need to expand the donor base. Given that recruitment costs range from €22 (US $25; via ambassador-driven efforts) to €58 (US $66; through direct, or “cold,” recruitment strategies), as reported in 2015 [32], a reduction in recruitment needs could significantly enhance the financial sustainability of blood donation organizations.

### Limitations and Strengths

Our study addresses a gap in existing research by examining the effects of naturalistic exposure to social media, closely mirroring real-world conditions. By utilizing an actual blood bank’s Facebook page, involving participants’ personal Facebook accounts, and collecting data through their daily routines, we enhanced the external validity of our findings. We also address the limitations of prior research by significantly expanding the sample size. Moreover, we extend the scope of this field by examining the potential effects of Facebook on attitudes over a longer duration, as well as investigating its impact on actual behavior.

However, several limitations should be noted. First, participants were aware of their involvement in the study, which may have introduced testing effects, such as increased attention to Facebook posts or altered attitudes and behaviors toward donation and the organization. Second, it is important to emphasize that the specific content and presentation style adopted by an organization on Facebook can significantly influence the outcomes. In media effects research, it is widely recognized that “content is king” [47]. However, our findings suggest that social media can positively influence blood donation behavior through approximately 4 weekly posts, which focus on the 5 key social media communication pillars of the blood bank: Donor Community, Better Patient Life, Inside the Blood Bank, Blood, and Plasma. Moreover, our previous research studies [8,11] provide additional insights into key issues that require attention, as well as the environment in which a blood bank’s social media communication competes.

Third, our study focused solely on Facebook to examine the impact of social media. However, recent trends show a shift among younger demographics away from Facebook toward platforms such as TikTok (ByteDance Ltd.) and Instagram (Meta Platforms, Inc.), which are gaining popularity across various age groups, including those aged 18 years and older—the demographic eligible for blood donation [48]. Consequently, expanding the range of social media platforms used in donor recruitment strategies could be more effective in reaching potential new donors. This approach is particularly relevant given the declining participation that may result from focusing solely on Facebook. However, it is important to note that individuals aged 29 years and above continue to engage with Facebook [48], which aligns closely with the average age of our study participants.

Fourth, we assessed each participant’s donation attempts by extracting this information from our database. However, it is important to note that donation intervals can vary based on the type of donation (eg, whole blood vs plasma), which could influence the total number of donations. Additionally, self-selection biases were present in our study. We had participants who were already positively inclined toward the blood bank and had followed the Facebook page before the study. Conversely, some participants in the control group chose to follow the page during the study. Fifth, some participants assigned to the experimental group opted not to participate. This self-selection could have led to an experimental group where participants with initially negative brand evaluations were underrepresented. Finally, it is important to recognize that our field trial did not account for all external factors. While we considered activity on other social media channels, we did not explore other potential influences, such as television and websites. However, the blood collection agency has a limited presence in television advertising or large-scale media campaigns, with its primary communication and promotional efforts concentrated on social media—particularly Facebook.

Future research should delve into the psychological and social mechanisms through which social media engagement influences blood donation behavior. Exploring mediating and moderating variables could offer a deeper understanding of how platforms such as Facebook impact donor attitudes and behaviors.

Practically speaking, we observed that many participants assigned to the experimental group who declined participation expressed reluctance to follow an organizational Facebook page immediately after registering as a blood donor but before making a donation. Based on these observations, we recommend that blood banks aiming to harness the potential impact of social media on donation behavior should actively invite new donors to join their social media pages only after they have completed their NDE or made their first donation.

### Conclusions

Our study provides initial evidence that regular engagement with a Facebook page can positively influence behavior, offering significant benefits to organizations, including blood banks. It demonstrates that social media efforts can be successful, even in the absence of strong attitudinal changes. Finally, we offer actionable insights and recommendations that blood banks and other organizations can adopt to replicate these results and encourage individuals to engage in beneficial actions.

## Appendix

Multimedia Appendix 1 Questionnaire and cross-sectional differences.

Multimedia Appendix 2 CONSORT 2010 checklist.

## Abbreviations

IRR: incidence rate ratio
NDE: New Donor Examination
OR: odds ratio

## Data Availability

The data sets generated and analyzed during this study are available from the corresponding author upon reasonable request.
