# Supplementary material for: Boosting Blood Donations Through Facebook Engagement: Randomized Controlled Field Trial
Source: J Med Internet Res. 2025 May 12;27:e64740. doi: 10.2196/64740 (PMC12107197; doi:10.2196/64740)
Supplement: Multimedia Appendix 1 [file jmir_v27i1e64740_app1.docx]

# Boosting Blood Donations Through Facebook Engagement: Randomized Controlled Field Trial

Steven Ramondt1,2, Peter Kerkhof2 & Eva-Maria Merz1,3

1 Donor Studies, Dept. of Donor Medicine Research, Sanquin Research, Amsterdam, the Netherlands

2 Dept. of Communication Science, Vrije Universiteit Amsterdam, the Netherlands

3 Center for Philanthropic Studies, Dept. of Sociology, Vrije Universiteit Amsterdam, the Netherlands

APPENDIX A Questionnaire

**Vragenlijst 1: de voormeeting**

1. **Attitude**

Hieronder staan zes rijen met aan beide kanten steeds twee uiterste begrippen. Per rij kunt u één van de vijf hokjes aankruisen. Zet per rij een kruisje in het hokje dat het meest op u van toepassing is.

Bloed/plasma geven vind ik:

|  | 1 | 2 | 3 | 4 | 5 |  |
| --- | --- | --- | --- | --- | --- | --- |
| Negatief |  |  |  |  |  | Positief |
| Goed |  |  |  |  |  | Slecht |
| Zinloos |  |  |  |  |  | Zinvol |
| Prettig |  |  |  |  |  | Onprettig |
| Vervelend |  |  |  |  |  | Leuk |
| Onaantrekkelijk |  |  |  |  |  | Aantrekkelijk |

1. **Intention**

Wilt u aangeven in hoeverre u het eens of oneens bent met de volgende stelling?

1 = helemaal mee oneens; 2= mee oneens; 3 = neutraal; 4 = mee eens; 5 = helemaal mee eens

|  | Helemaal mee oneens (1) |  |  |  | Helemaal mee eens (5) |
| --- | --- | --- | --- | --- | --- |
| Ik ben van plan om bloed/plasma te blijven geven, zolang mijn gezondheid dat toestaat |  |  |  |  |  |
| Ik blijf bloed/plasma donor totdat ik niet meer mag doneren |  |  |  |  |  |

1. **Brand attitude**

Wat is uw indruk van Sanquin? Wilt u op een schaal van 1 tot 9 aangeven wat u van Sanquin vindt?

| Slecht (1) |  |  |  |  |  |  |  | Goed (9) |
| --- | --- | --- | --- | --- | --- | --- | --- | --- |
| Niet leuk (1) |  |  |  |  |  |  |  | Leuk (9) |
| Onaantrekkelijk (1) |  |  |  |  |  |  |  | Aantrekkelijk (9) |
| Kwalitatief slecht (1) |  |  |  |  |  |  |  | Kwalitatief goed (9) |

1. **Conversational human voice**

Wilt u op een schaal van 1 tot 7 aangeven in hoeverre u het eens of oneens bent met de volgende stellingen? In mijn opinie, Sanquin…

|  | Helemaal niet mee eens (1) |  |  |  |  |  | Helemaal mee eens (7) |
| --- | --- | --- | --- | --- | --- | --- | --- |
| nodigt mensen uit tot conversatie |  |  |  |  |  |  |  |
| staat open voor dialoog |  |  |  |  |  |  |  |
| communiceert alsof we een gesprek voeren |  |  |  |  |  |  |  |
| probeert te communiceren met een menselijke stem |  |  |  |  |  |  |  |
| probeert interessant te communiceren |  |  |  |  |  |  |  |
| gebruikt humor in communicatie |  |  |  |  |  |  |  |
| probeert communicatie aangenaam te maken |  |  |  |  |  |  |  |
| zou een fout toegeven |  |  |  |  |  |  |  |
| levert snelle feedback op kritiek in een directe manier zonder kritisch te zijn |  |  |  |  |  |  |  |
| behandelt mij en anderen als mens |  |  |  |  |  |  |  |

1. **Warmth & competence**

Ik vind dat Sanquin…

|  | Helemaal niet mee eens (1) |  |  |  |  |  | Helemaal mee eens (7) |
| --- | --- | --- | --- | --- | --- | --- | --- |
| Warm is |  |  |  |  |  |  |  |
| Vrijgevig is |  |  |  |  |  |  |  |
| Vriendelijk is |  |  |  |  |  |  |  |
| Competent is |  |  |  |  |  |  |  |
| Efficiënt is |  |  |  |  |  |  |  |
| Effectief is |  |  |  |  |  |  |  |

1. **Contemplation**

Hoe vaak denkt u aan bloed doneren?

1 = Nooit

2 = Eens per jaar

3 = Een paar keer per jaar

4 = Eens elke twee maanden

5 = Eens per maand

6 = Eens per week

7 = Een paar keer per week

8 = Eens per dag

9 = Een paar keer per dag

Hoe vaak denkt u aan Sanquin?

1 = Nooit

2 = Eens per jaar

3 = Een paar keer per jaar

4 = Eens elke twee maanden

5 = Eens per maand

6 = Eens per week

7 = Een paar keer per week

8 = Eens per dag

9 = Een paar keer per dag

1. **General Facebook time**

Hoe vaak zit u op Facebook?

1 = Nooit

2 = Eens per jaar

3 = Een paar keer per jaar

4 = Eens elke twee maanden

5 = Eens per maand

6 = Eens per week

7 = Een paar keer per week

8 = Eens per dag

9 = Een paar keer per dag

10 = Eens elke 2 uur

11 = Eens per uur

12 = Een paar keer per uur

1. **General Facebook Intensity**

Wilt u op een schaal van 1 (=helemaal niet mee eens) tot 5 (=helemaal mee eens) aangeven in hoeverre u het eens of oneens bent met de volgende stellingen?

|  | Helemaal niet mee eens (1) |  |  |  |  |  | Helemaal mee eens (5) |
| --- | --- | --- | --- | --- | --- | --- | --- |
| Facebook is onderdeel van mijn dagelijkse activiteit |  |  |  |  |  |  |  |
| Ik vertel met trots dat ik op Facebook zit |  |  |  |  |  |  |  |
| Facebook is onderdeel geworden van mijn dagelijkse routine |  |  |  |  |  |  |  |
| Ik voel me vreemd als ik een tijdje niet op Facebook ben ingelogd |  |  |  |  |  |  |  |
| Ik voel me onderdeel van de Facebook gemeenschap |  |  |  |  |  |  |  |
| Ik zou het jammer vinden als Facebook stopt. |  |  |  |  |  |  |  |

1. **Facebook exposure**

Hoe vaak heeft u in de vorige maand de Facebook pagina van Sanquin bezocht?

1 = Nooit

2 = 1-3 keer

3 = 4-6 keer

4 = 7-9 keer

5 = 10-12 keer

6 = 12-15 keer

7 = meer dan 15 keer

Hoe vaak heeft u in de vorige maand Facebook berichten gezien van Sanquin?

1 = Nooit

2 = 1-3 keer

3 = 4-6 keer

4 = 7-9 keer

5 = 10-12 keer

6 = 12-15 keer

7 = meer dan 15 keer

1. Bent u lid van de Sanquin Facebook pagina doordat u de pagina heeft ‘geliked’?

Ja (1) / Nee (0)

**Als nee ->Randomisatie naar controle conditie, standaard nieuwe volger, en uitbreide nieuwe volger. Elk persoon krijgt een groepscode (bijvoorbeeld ‘1’ voor de controle groep) toegewezen voor identificatie tijdens de nameting**

**Standaard nieuwe volger: ga door naar vraag 12**

**Uitgebreide nieuwe volger: ga door naar vraag 15**

**Controle conditie: ga door naar vraag 18**

1. Hoelang bent u al lid van de Sanquin Facebook pagina?

1 = Minder dan 1 week

2 = 1-3 weken

3 = 1-2 Maanden

4 = 3-6 Maanden

5 = 6-12 Maanden

6 = 1-2 Jaar

7 = meer dan 2 jaar

**Ga door naar vraag 18**

1. Voor dit onderzoek is het belangrijk dat u de Sanquin Facebook pagina volgt door de pagina te ‘liken’. Over twee maanden wordt u uitgenodigd om hierover een aantal vragen in te vullen.

Zou u de Sanquin Facebook pagina willen liken? Voor dit onderzoek is het van belang dat u direct de Sanquin Facebook pagina liked. Dit kan doormiddel van de onderstaande link:

LINK

**Volgende pagina**

1. Heeft u de pagina geliked? Ja (1) / Nee (0)

**Als ja ->ga door naar vraag 18**

1. Waarom heeft u de pagina niet geliked? _____

**Ga door naar vraag 18**

1. Voor dit onderzoek is het belangrijk dat u de Sanquin Facebook pagina volgt door de pagina te ‘liken’ via de link onder dit bericht. Over twee maanden wordt u uitgenodigd om hierover een aantal vragen in te vullen.

Zou u de Sanquin Facebook pagina willen liken via de onderstaande link? Voor dit onderzoek is het van belang dat u direct de Sanquin Facebook pagina liked.

LINK

**Volgende pagina**

1. Heeft u de pagina geliked? Ja (1) / Nee (0)

**Als ja ->ga door naar vraag 18**

1. Waarom heeft u de pagina niet geliked? _____
2. Wat is uw geslacht?

0 = man

1 = vrouw

2 = anders

3 = geen antwoord

1. Wat is uw burgerlijke staat?

1 = Ongehuwd

2 = Gehuwd of geregistreerd partnerschap of partner?

3 = Gescheiden

4 = Weduwe / weduwnaar

1. Heeft u kinderen? Ja (1)/ Nee (0)

**Als nee -> ga door naar vraag 20**

1. Hoeveel kinderen heeft u? (In getallen, bijvoorbeeld : 2) ____
2. Wat is de hoogste schoolopleiding die u met een diploma heeft afgerond?

1 = Geen

2 = Lager onderwijs (basisonderwijs)

3 = Lager beroepsonderwijs (VMBO, LBO: bijv. LTS, LAS, LHNO, VBO, LEAD)

4 = Middelbaar voortgezet onderwijs bijv. ULO, MULO, MAVO, LAVO, VGLO)

5 = Hoger voortgezet onderwijs (bijv. HAVO, VWO, MMS, HBS, atheneum, gymnasium)

6 = Hoger beroepsonderwijs (HBO: bv. HTS, HAS, HEAO, PABO)

7 = Universiteit

1. Tot welke groep(en) behoort u? (meerdere antwoorden mogelijk)

1 = Ik werk (loondienst, zelfstandig ondernemer, zzp’er)

2 = Ik ben met (vervroegd) pensioen (AOW, VUT, FPU, OBU)

3 = Ik zit in de Ziektewet (ZW)

4 = Ik ben werkloos/werkzoekend (WW, WWB)

5 = Ik ben arbeidsongeschikt (WIA (WAO), AAW, WAZ, WAJONG)

6 = Ik ben huisvrouw/huisman

7 = Ik ben student/scholier met een bijbaan

8 = Ik ben student/scholier zonder bijbaan

9 = Anders

1. Wat is uw etnische achtergrond? ( Heeft u ouders met een verschillende etnische achtergrond? Kruis dan meerdere antwoorden aan)

1 = Nederlands

2 = Surinaams

3 = Antilliaans

4 = Marokkaans

5 = Turks

6 = Afrikaans (anders dan Marokkaans)

7 = Aziatisch (anders dan Turks)

8 = Arabisch

9 = Indonesisch

10 = Anders

____________________

**Vragenlijst 2 & 3: de nameting**

1. Bent u bekend met de Facebook pagina van Sanquin? Ja (1) / Nee (0)

**Als nee -> (ga door naar vraag 7)**

**Als ja,**

**Controle en huidige volger groep: ga door naar vraag 2**

**Standaard nieuwe & uitgebreide nieuwe volger: ga door naar vraag 3**

1. Bent u lid van de Sanquin Facebook pagina doordat u de pagina heeft ‘geliked’? Ja (1) / Nee (0)

**Als nee -> (skip naar vraag 7)**

**Als ja -> (ga door naar vraag 5**

1. **Standaard nieuwe volger & uitgebreide nieuwe volger manipulatie check**

Heeft de Facebook pagina van Sanquin ‘geliked’ toen we dat aan u vroegen in de eerste survey?

**Als nee -> (ga door naar vraag 7)**

1. Liked u de Facebook pagina van Sanquin nog steeds?

1 = Ja

2 = Nee, ik heb de pagina de dag van de eerste survey ‘geunliked’

3 = Nee, ik heb de pagina na een paar dagen ‘geunliked’

4 = Nee, ik heb de pagina na 1 tot 2 weken ‘geunliked’

5 = Nee, ik heb de pagina na 3 tot 4 weken ‘geunliked’

6 = Nee, ik heb de pagina afgelopen maand ‘geunliked’

**Als 2 - 6 -> (ga door naar vraag 7)**

1. **Facebook exposure**

Hoe vaak heeft u in de vorige maand de Facebook pagina van Sanquin bezocht?

1 = Nooit

2 = 1-3 keer

3 = 4-6 keer

4 = 7-9 keer

5 = 10-12 keer

6 = 12-15 keer

7 = meer dan 15 keer

Hoe vaak heeft u in de vorige maand Facebook berichten gezien van Sanquin?

1 = Nooit

2 = 1-3 keer

3 = 4-6 keer

4 = 7-9 keer

5 = 10-12 keer

6 = 12-15 keer

7 = meer dan 15 keer

1. **Facebook attitude T_2_**

Wilt u op een schaal van 1 (=helemaal niet mee eens) tot 5 (=helemaal mee eens) aangeven in hoeverre u het eens of oneens bent met de volgende stellingen?

|  | Helemaal niet mee eens (1) |  |  |  |  |  | Helemaal mee eens (5) |
| --- | --- | --- | --- | --- | --- | --- | --- |
| Ik zou de pagina van Sanquin graag blijven volgen |  |  |  |  |  |  |  |
| Ik ben tevreden met de inhoud van de Facebook pagina van Sanquin |  |  |  |  |  |  |  |
| Ik voel mij comfortabel in het volgen van de Facebook pagina van Sanquin |  |  |  |  |  |  |  |
| Ik vind dat het bekijken van de Facebook pagina/post van Sanquin een goede manier is van besteden van mijn tijd |  |  |  |  |  |  |  |
| In vergelijking met andere Facebook pagina’s die ik like, vind ik de Facebook pagina van Sanquin één van de beste |  |  |  |  |  |  |  |

|  | Helemaal niet leuk (1) |  |  |  |  |  | Heel leuk (10) |
| --- | --- | --- | --- | --- | --- | --- | --- |
| Hoe leuk vond u de Facebook pagina van Sanquin |  |  |  |  |  |  |  |
| Hoe leuk vond u de Facebook berichten van Sanquin |  |  |  |  |  |  |  |

1. **Attitude**

Hieronder staan zes rijen met aan beide kanten steeds twee uiterste begrippen. Per rij kunt u één van de vijf hokjes aankruisen. Zet per rij een kruisje in het hokje dat het meest op u van toepassing is.

Bloed / plasma geven vind ik:

|  | 1 | 2 | 3 | 4 | 5 |  |
| --- | --- | --- | --- | --- | --- | --- |
| Negatief |  |  |  |  |  | Positief |
| Goed |  |  |  |  |  | Slecht |
| Zinloos |  |  |  |  |  | Zinvol |
| Prettig |  |  |  |  |  | Onprettig |
| Vervelend |  |  |  |  |  | Leuk |
| Onaantrekkelijk |  |  |  |  |  | Aantrekkelijk |

1. **Intention**

Wilt u aangeven in hoeverre u het eens of oneens bent met de volgende stelling?

|  | Helemaal mee oneens (1) |  |  |  | Helemaal mee eens (5) |
| --- | --- | --- | --- | --- | --- |
| Ik ben van plan om bloed / plasma te blijven geven, zolang mijn gezondheid dat toestaat |  |  |  |  |  |
| Ik blijf bloed-/plasma donor totdat ik niet meer mag doneren |  |  |  |  |  |

1. **Brand attitude**

Wat is uw indruk van Sanquin? Wilt u op een schaal van 1 tot 9 aangeven wat u van Sanquin vindt?

| Slecht (1) |  |  |  |  |  |  |  | Goed (9) |
| --- | --- | --- | --- | --- | --- | --- | --- | --- |
| Niet leuk (1) |  |  |  |  |  |  |  | Leuk (9) |
| Onaantrekkelijk (1) |  |  |  |  |  |  |  | Aantrekkelijk (9) |
| Kwalitatief slecht (1) |  |  |  |  |  |  |  | Kwalitatief goed (9) |

1. **Conversational human voice**

Wilt u op een schaal van 1 tot 7 aangeven in hoeverre u het eens of oneens bent met de volgende stellingen? In mijn opinie, Sanquin…

|  | Helemaal niet mee eens (1) |  |  |  |  |  | Helemaal mee eens (7) |
| --- | --- | --- | --- | --- | --- | --- | --- |
| nodigt mensen uit tot conversatie |  |  |  |  |  |  |  |
| Sanquin staat open voor dialoog |  |  |  |  |  |  |  |
| communiceert alsof we een gesprek voeren |  |  |  |  |  |  |  |
| probeert te communiceren met een menselijke stem |  |  |  |  |  |  |  |
| probeert interessant te communiceren |  |  |  |  |  |  |  |
| gebruikt humor in communicatie |  |  |  |  |  |  |  |
| probeert communicatie aangenaam te maken |  |  |  |  |  |  |  |
| zou een fout toegeven |  |  |  |  |  |  |  |
| levert snelle feedback op kritiek in een directe manier zonder kritisch te zijn |  |  |  |  |  |  |  |
| behandelt mij en anderen als mens |  |  |  |  |  |  |  |

1. **Warmth & competence**

Ik vind dat Sanquin…

|  | Helemaal niet mee eens (1) |  |  |  |  |  | Helemaal mee eens (7) |
| --- | --- | --- | --- | --- | --- | --- | --- |
| Warm is |  |  |  |  |  |  |  |
| Vrijgevig is |  |  |  |  |  |  |  |
| Vriendelijk is |  |  |  |  |  |  |  |
| Competent is |  |  |  |  |  |  |  |
| Efficiënt is |  |  |  |  |  |  |  |
| Effectief is |  |  |  |  |  |  |  |

1. **Contemplation**

Hoe vaak denkt u aan bloed doneren?

1 = Nooit

2 = Eens per jaar

3 = Een paar keer per jaar

4 = Eens elke twee maanden

5 = Eens per maand

6 = Eens per week

7 = Een paar keer per week

8 = Eens per dag

9 = Een paar keer per dag

Hoe vaak denkt u aan Sanquin?

1 = Nooit

2 = Eens per jaar

3 = Een paar keer per jaar

4 = Eens elke twee maanden

5 = Eens per maand

6 = Eens per week

7 = Een paar keer per week

8 = Eens per dag

9 = Een paar keer per dag

1. **General Facebook time**

Hoe vaak zit je op Facebook?

1 = Nooit

2 = Eens per jaar

3 = Een paar keer per jaar

4 = Eens elke twee maanden

5 = Eens per maand

6 = Eens per week

7 = Een paar keer per week

8 = Eens per dag

9 = Een paar keer per dag

10 = Eens elke 2 uur

11 = Eens per uur

12 = Een paar keer per uur

1. **General Facebook Intensity**

Wilt u op een schaal van 1 (=helemaal niet mee eens) tot 5 (=helemaal mee eens) aangeven in hoeverre u het eens of oneens bent met de volgende stellingen?

|  | Helemaal niet mee eens (1) |  |  |  |  |  | Helemaal mee eens (5) |
| --- | --- | --- | --- | --- | --- | --- | --- |
| Facebook is onderdeel van mijn dagelijkse activiteit |  |  |  |  |  |  |  |
| Ik vertel met trots dat ik op Facebook zit |  |  |  |  |  |  |  |
| Facebook is onderdeel geworden van mijn dagelijkse routine |  |  |  |  |  |  |  |
| Ik voel me vreemd als ik een tijdje niet op Facebook ben ingelogd |  |  |  |  |  |  |  |
| Ik voel me onderdeel van de Facebook gemeenschap |  |  |  |  |  |  |  |
| Ik zou het jammer vinden als Facebook stopt. |  |  |  |  |  |  |  |

APPENDIX B Cross-sectional differences

For H2, we expected both current and new blood bank Facebook followers to have more positive attitudes and higher intentions to donate blood compared to non-followers. The analyses do indeed show differences in attitude towards blood donation, intention to donate blood, attitude towards the blood bank, and blood bank contemplation between the groups after two months (resp. F (2, 1159) = 9.52, p < .001; F (2, 1159) = 3.10, p = .045; F (2, 1159) = 4.42, p = .012; F (2, 968) = 4.46, p = .012). Yet, Bonferroni pairwise comparisons show that the higher and more positive values only apply to the observational group. In contrast with H2, we do not see higher or more positive values of attitudes intentions, and contemplation regarding the blood bank and donating for participants in the experimental group (relative to the control group). Comparisons show a more positive attitude toward blood donation for the observational compared to the control group (p = .002) and experimental group (p < .001), more positive attitudes towards the blood bank for the observational group compared with the control group (p = .026) and experimental group (p = .020) for. For intention to donate blood and blood bank contemplation, Bonferroni pairwise comparisons show higher scores for the observational compared with the control group only (resp. p = .035; p = .010). No differences exist between donorship contemplation, warmth, and competence (resp. F (2, 1159) = .54, p = .584; F (2, 1159) = 1.90, p = .149; F (2, 1159) = .23, p = .793).

The differences after two months largely dissipate after one year. In contrast with H2, we no longer see differences between the groups after one year for attitudes towards blood donation, intention to donate, and attitude towards the blood bank (resp. F (2, 413) = 1.36, p = .257; F (2, 413) = 1.13, p = .323; F (2, 413) = 1.48, p = .229). In addition, no differences after one year are found for warmth F (2, 413) = .32, p =.730, competence F (2, 413) = .07, p = .935, and contemplation regarding donorship and the blood bank F (2, 413) = .42, p = .657. Only for contemplation about the blood bank we find differences between groups after one year, F (2, 321) = 3.23, p = .041 (see Table 3 for means (SDs)). Bonferroni pairwise comparisons show that the observational group contemplates more about the blood bank compared to the control group (p = .033).

**Table B1. Main attitudinal outcomes after two months.**

| Depend. variables | Experiment (n = 393) | | |  | Control (n = 496) | | |  | Observational (n = 273) | | |
| --- | --- | --- | --- | --- | --- | --- | --- | --- | --- | --- | --- |
|  | Pre | post | p |  | Pre | post | p |  | Pre | post | p |
| Attitude blood donation | 4.26 (.43) | 4.16 (.40) | <.001 |  | 4.17 (.43) | 4.19 (.45) | .326 |  | 4.28 (.48) | 4.31 (.48) | .377 |
| Intention blood donation | 4.36 (.81) | 4.22 (.86) | .005 |  | 4.29 (.82) | 4.17 (.87) | .011 |  | 4.42 (.77) | 4.33 (.81) | .158 |
| Attitude Blood bank | 7.49 (1.02) | 6.98 (1.23) | <.001 |  | 7.33 (1.12) | 7.00 (1.23) | <.001 |  | 7.59 (1.09) | 7.25 (1.39) | <.001 |
| Warmth | 5.59 (.84) | 5.03 (.98) | <.001 |  | 5.44 (.92) | 5.09 (.97) | <.001 |  | 5.59 (.99) | 5.18 (1.14) | <.001 |
| Competence | 5.85 (.90) | 5.33 (1.07) | <.001 |  | 5.73 (.94) | 5.38 (1.03) | <.001 |  | 5.76 (1.07) | 5.37 (1.18) | <.001 |
| Contemplation donorship | 4.08 (1.32) | 3.83 (1.21) | <.001 |  | 4.04 (1.16) | 3.82 (1.27) | <.001 |  | 3.96 (1.35) | 3.80  (1. 39) | .094 |
| Contemplation Blood bank | 3.31 (1.62) | 3.95 (1.46) | <.001 |  | 3.28 (1.6) | 3.78 (1.43) | <.001 |  | 3.79 (1.66) | 4.14 (1.56) | .001 |

**Table B2. Main attitudinal outcomes after a year.**

| Depend. variables | Experiment (n = 151) | | |  | Control (n = 158) | | |  | Observational (n = 107) | | |
| --- | --- | --- | --- | --- | --- | --- | --- | --- | --- | --- | --- |
|  | Pre | post | p |  | Pre | post | p |  | Pre | post | p |
| Attitude blood donation | 4.37 (.50) | 4.22 (.37) | <.001 |  | 4.26 (.47) | 4.19 (.47) | .093 |  | 4.45 (.43) | 4.28 (.47) | <.001 |
| Intention blood donation | 4.60 (.70) | 4.26 (.73) | <.001 |  | 4.46 (.76) | 4. 22 (.79) | .002 |  | 4.70 (.55) | 4.36 (.89) | <.001 |
| Attitude blood bank | 7.63 (1.06) | 7.13 (1.19) | <.001 |  | 7.54 (1.08) | 7.06 (1.30) | <.001 |  | 7.76 (1.01) | 7.33 (1.33) | <.001 |
| Warmth | 5.72 (.74) | 5. 07 (.92) | <.001 |  | 5.69 (.84) | 5. 08 (1.01) | <.001 |  | 5.77 (.78) | 5.16 (1.06) | <.001 |
| Competence | 5.89 (.81) | 5.37 (1.02) | <.001 |  | 5.84 (.82) | 5.36 (1.06) | <.001 |  | 6.02 (.72) | 5.41 (1.18) | <.001 |
| Contemplation donorship | 4.15 (1.32) | 3.90 (1.37) | .062 |  | 3.75 (1.13) | 3.75 (1.27) | 1.000 |  | 4.10 (1.21) | 3.72 (1.41) | .015 |
| Contemplation blood bank | 3.53 (1.61) | 3.94 (1.35) | .005 |  | 3.41 (1.50) | 3.71 (1.26) | .026 |  | 3.47 (1.59) | 4.18 (1.40) | <.001 |
